# Digital Mental Health: Towards Personalised Care in Psychiatry

**DOI:** 10.1192/j.eurpsy.2022.42

**Published:** 2022-09-01

**Authors:** I. Myin-Germeys

**Affiliations:** KU Leuven, Neurosciences, Center For Contextual Psychiatry, Leuven, Belgium

## Abstract

Within psychiatry, there is a need for more personalized and person-centered care. Whereas the focus has largely been on the search for biomarkers to advance the field, I will focus on the use of digital technology to further personalized care in psychiatry. I will discuss how using a structured diary applet could help in developing a much more fine-grained understanding of how psychopathology emerges in the realm of ordinary life, often in interaction with contextual factors. This digital mobile health tool makes patients active partners in the clinical process as they will have to actively collect their own data. This will not only increase empowerment and self-management, it also will allow to provide more detailed evaluations of both medical and psychological interventions, it will point towards individualized treatment goals as well as provide a tool for shared decision making. In addition, I will discuss the possibility of digital mobile health tools for blended care, where standard clinical therapeutic interventions are extended outside the therapy room into the daily life of people. I will give some examples of such Ecological Momentary Interventions, where digital tools are used to offer psychological intervention in daily life, with a specific focus on young people.

**Disclosure:**

No significant relationships.

